# Assessing the quality of postnatal care offered to mothers and babies by midwives in Lilongwe District

**DOI:** 10.4102/safp.v62i1.5026

**Published:** 2020-07-23

**Authors:** Mercy Pindani, Chrissie Phiri, Wanangwa Chikazinga, Idesi Chilinda, Janet Botha, Genesis Chorwe-Sungani

**Affiliations:** 1 Department of Community Health, Faculty of Community Health Studies, Kamuzu College of Nursing, Lilongwe, Malawi; 2 Department of Midwifery, Faculty of Maternal, Neonatal and Reproductive Health Studies, Kamuzu College of Nursing, Lilongwe, Malawi; 3 Department of Education and Communication Studies, Faculty of Applied Health Studies, Kamuzu College of Nursing, Lilongwe, Malawi; 4 Department of Mental Health, Faculty of Community Health Studies, Kamuzu College of Nursing, Blantyre, Malawi

**Keywords:** postnatal care, quality, midwives, babies

## Abstract

**Background:**

The quality of care received by mothers and newborns in low-resource settings is often poor. This may partly explain the high rates of maternal deaths (60%) that occur during the postpartum period in Malawi. However, the quality of care provided to mothers and newborns in the country has not been adequately assessed. Therefore, this study aimed at assessing the quality of postnatal care services offered to mothers and babies by midwives in Lilongwe District.

**Methods:**

This was a quantitative study that used a sample of 58 midwives to assess the quality of postnatal care at three selected health facilities. A structured questionnaire, an observation tool and a facility checklist were used to collect data. Descriptive statistics were used to analyse the data. The study received ethics approval from the relevant authority.

**Results:**

The study found that the percentages reported by midwives regarding client monitoring varied and were below the 80% threshold. Midwives did not always follow the reproductive health standards on client examination so that less than 75% of midwives inspected perineal wounds (52.2%), checked vital signs of neonate (66.7%) and mother (62.2%), and inspected lochia drainage (30.4%). Most midwives (91.3%) never assessed the emotional state of the mother. Midwives covered a range of topics during health education and counselling. However, some topics, including immunisations (31.1%), were never taught.

**Conclusion:**

The study has suggested that the postnatal care offered by midwives at three health facilities was generally substandard and midwives do not always monitor, assess and counsel postnatal clients.

## Introduction

Postpartum care is a vital part of the childbearing process; accordingly, midwives are obliged to provide quality care for the mother and baby from birth up to 6 weeks postnatal. However, the quality of care received by mothers and newborns in low-resource settings is often poor.^1^ This is collaborated by Nesbitt and colleagues who found that the quality of routine and emergency intrapartum and postnatal care was generally poor.^2^ However, this is taking place despite the existence of the World Health Organization (WHO) guidelines, which recommend the provision of quality postnatal care to mothers and newborns.^3^ It is widely acknowledged that quality of care includes multiple levels from patient to health system, and other dimensions such as safety and efficiency.^1^ Nevertheless, quality of care is difficult to measure.^2^ In this study, quality of care refers to ‘the degree to which postnatal services for individuals and populations increase the likelihood of timely and appropriate treatment for the purpose of achieving desired outcomes that are both consistent with current professional knowledge and uphold basic reproductive rights.’ (p. 9)^1^ Quality of care helps in reducing maternal and newborn deaths^4^ and is a key predictor of the utilisation of postnatal care services in Malawi.^5^

In Malawi, one study found that the quality of postnatal care services was below standard in one of the districts.^6^ It is evident that some midwives do not follow stipulated guidelines in the management of postpartum women in the country,^6^ which negatively affects the quality of care received by mothers and their newborns. It has been documented that adhering to clinical practice guidelines may improve midwives’ knowledge, clinical skills and attitudes when providing postnatal care.^7^ Literature suggests that a ‘skilled attendant’ is essential in the provision of quality postnatal care to mothers and newborns.^1^ However, a shortage of skilled staff is a major obstruction to the provision of timely and quality postnatal care in Malawi.^8^ Furthermore, inflexible scheduling and staff allocations make it difficult to deliver quality postnatal care in the country,^8^ including emergency obstetric care (EmOC) and basic emergency obstetric and newborn care (BEmONC).

The EmOC and BEmONC signal functions and interventions that treat the causes of maternal and neonatal deaths are used to measure the quality of postnatal care in Malawi. As such, midwives working in emergency obstetric and newborn care (EmONC) and BEmONC facilities in Malawi are trained to offer essential postpartum care although they provide postnatal care of poor quality.^8^ There is a high maternal mortality ratio in Malawi (439 deaths per 100 000),^9^ which may partly be attributed to the substandard quality of EmONC in some facilities in the country.^6^ This is corroborated by Filby and colleagues who assert that the high maternal and neonatal mortality observed in low-resource settings may be due to a lack of quality postnatal care.^4^ The situation may be dire in Malawi where 2% of EmONC facilities meet the basic requirements for the provision of quality postnatal care.^6^ This shows that the quality of care provided to mothers and newborns in Malawi is given limited attention and has not been adequately assessed from the care provider’s perspective. Therefore, the aim of this study was to assess the quality of postnatal care services offered to mothers and babies by midwives in Lilongwe District.

## Materials and methods

### Study design

This was a descriptive quantitative study that assessed the quality of postnatal care provided by midwives in Lilongwe District, Malawi. The study focused on the structural, process and outcome of postnatal care.

### Study setting

The study was conducted in BEmONC and EmONC facilities located in semi-rural (Lumbadzi Health Centre) and urban areas (Bwaila and Kawale health centres) in Lilongwe District, Malawi. These facilities offer maternal and neonatal health services and had midwives trained in EmONC and BEmONC. Further details of the settings are described in Table 1^10^.

**TABLE 1:** Description of study settings.

Health facility	Population served	Women of child-bearing age	Expected deliveries in a year
Bwaila Hospital	177 617	40 852	8881
Kawale Health Centre	268 827	61 830	13 441
Lumbadzi Health Centre	84 008	19 322	4200

*Source*: Ministry of Health. Central West Zone performance report 2013/2014. Lilongwe: Ministry of Health; 2015

### Study population

The target population for the study were all enrolled nurse midwives, nurse midwife technicians and registered midwives (*N* = 91) working in maternity units (labour ward, postnatal ward and nursery) from the three health facilities (Bwaila, *n* = 66; Lumbadzi, *n* = 13; and Kawale, *n* = 12). Enrolled nurse midwives and nurse midwife technicians are the lowest cadre of midwives, with a college Certificate or Diploma in Nursing and Midwifery, while registered midwives have either a Bachelor of Science in Nursing and Midwifery or a Bachelor of Science in Nursing, in addition to a university Certificate in Midwifery, a Diploma in Nursing and Midwifery or a Diploma in Nursing plus a university Certificate in Midwifery.^11^

### Sample size

The study intended to use census (all 91 midwives). However, 58 midwives (Bwaila, *n* = 48; Lumbadzi, *n* = 4; Kawale, *n* = 6), representing 64% of the target population, participated in this study. Some midwives (Bwaila, *n* = 4; Lumbadzi, *n* = 5; Kawale, *n* = 3) declined to participate in the study. All midwives of different cadres working full-time in the labour, postnatal and neonatal nursery wards during the data collection period (2016) were included in this study; those working relief duty and as intern midwives were excluded.

### Instruments

A structured questionnaire, an observation tool and a facility checklist were used to collect the data. The tools were designed in line with the Malawi Ministry of Health Integrated Maternal and Neonatal Care guidelines^3^ and WHO postnatal guidelines. The structured questionnaire for midwives had four sections focusing on demographic data, knowledge of midwives, practices of midwives, and attitudes of midwives during the provision of postnatal care. A Likert scale ranging from 1 (strongly disagree) to 5 (strongly agree) was used to assess the attitudes of midwives. The structured observation tool was used to observe the actual practices of midwives during the provision of postnatal care. The focus was on the availability of resources; care provision; the collection of subjective data; the collection of objective data; management of minor ailments; and health education and counselling. The facility checklist focused on the availability of human and material resources, infrastructure and some practices of midwives in the provision of postnatal care. The developed tools were reviewed by experts in the Maternal and Child Health (MCH) and Community and Mental Health departments (CMH) of the University of Malawi and the Kamuzu College and pretested on midwives at the Area 18 Health Centre.

### Data collection procedure

The researchers personally invited the midwives to participate in the study and those who accepted gave written consent. The researchers distributed a total of 58 self-administered questionnaires to participants (Bwaila, *n* = 48; Lumbadzi, *n* = 4; Kawale, *n* = 6) and collected 58 completed questionnaires, checking them to ensure there were no missing data and resulting in a response rate of 100%.

The researchers also conducted structured observations of actual practices of 30 out of the 58 midwives who answered the questionnaires (Bwaila, *n* = 20; Kawale, *n* = 6; Lumbadzi, *n* = 4) during the provision of postnatal care to mothers and babies. The observations included client monitoring, physical examinations, the management of minor ailments, and client education and counselling. This was done in the labour ward in the first 2 hours after delivery and in the postnatal ward until discharge. In addition, using a checklist, the researchers assessed the facilities to determine the availability of human and material resources for the provision of maternal and neonatal postnatal care.

### Data analysis

Data were entered into the Statistical Package for Social Sciences (SPSS version 22.0) and were then analysed by generating descriptive statistics (frequencies, percentages) and graphs. Scores on the outcome variables were computed as percentages and later compared to the cut-off point of 80% for the facility deemed to provide quality care according to the 20-criteria checklist derived from the Reproductive Health (RH) standards and WHO guidelines.

### Ethical consideration

Ethical approval was obtained from the College of Medicine Research and Ethics Committee, University of Malawi, NORHED 001/12/2014.

## Findings

### Cadres of midwives

This study showed that midwife–client ratios were very high (> 1:200) in all health facilities (Table 2). Nearly two thirds of midwives (60%, *n* = 27) in this study were nurse-midwife technicians (Table 2).

**TABLE 2:** Cadre of midwives and midwife to client ratio for each health facility.

Health facility	Cadre of staff			Total	Midwife to client ratio
	Enrolled nurse or midwife	Nurse or midwife technician	Registered nurse or midwife		
Bwaila Hospital	2	24	9	35	1:421
Kawale health centre	2	2	2	6	1:219
Lumbadzi health centre	2	1	1	4	1:327
**Total (*n*)**	**6**	**27**	**12**	**45**	**-**
**Total (%)**	**13.3**	**60.0**	**26.7**	**100.0**	**-**

#### Physical infrastructure

The study found that the EmONC facility (Bwaila Hospital) and the BEmONC facilities (Kawale and Lumbadzi health centres) had a postnatal ward and examination rooms with a couch. The infrastructure in all three facilities was in a good condition for safe care.

#### Equipment, drugs and supplies

**FIGURE 1:**
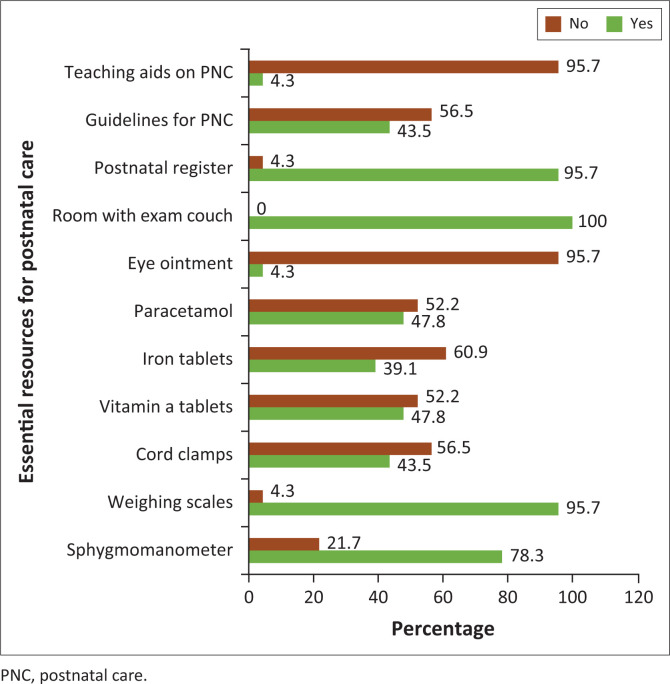
Availability of essential resources for postnatal care.

Midwives reported a varied availability of selected resources in health facilities, with a postnatal register, room with an examination couch and weighing scale registering the highest availability (> 80%). The least available resources were teaching aids for postnatal care and eye ointment (< 5%) (Figure 1).

### Quality of postnatal care services provided by midwives

#### Client monitoring

The percentages reported by midwives regarding client monitoring varied and were below the 80% threshold in this study. More than one third of midwives (37.8%) monitored the mothers’ condition every 15 minutes during the fourth stage of labour; another third (33.3%) monitored the mother every 30 min; and more than a quarter of midwives (28.9%) monitored the mother only once. Nearly two thirds of the midwives (60.9%) conducted the subsequent comprehensive assessment of the neonate 1 h after birth, 21.7% assessed the neonate after 2 h or more and the balance of the midwives (17.4%) did the assessment on discharge. There were also some disparities between the midwives’ self-reports and their actual practice. More than half of the midwives (55.6%) reported that they monitored and assessed the mother and baby in the postnatal ward twice a day, 20% indicated once a day and 24.2% only during discharge. However, the structured observation of their actual practice revealed that 65.2% of the midwives assessed the mother and the neonate once a day, 30.4% did this on discharge and only 4.4% monitored and assessed the mother and neonate twice a day.

#### Client examination

The study found that midwives did not always follow the RH standards on client examination, with many aspects scoring below 80%. Many of the midwives (57.8%) kept the mother in the labour ward for 2 h after delivery in order for them to identify complications early, while 42.2% kept the mother in the labour ward for less than 1 h after delivery. Furthermore, this study showed that postnatal women were observed for 24 h or less by nearly three quarters of the midwives (73.3%) compared to 26.7% who observed postnatal mothers for 48 h.

**FIGURE 2:**
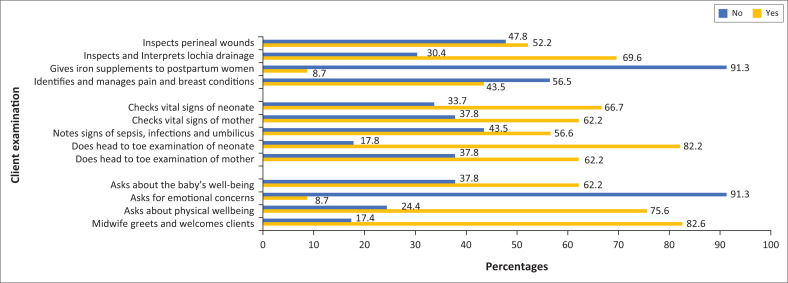
Midwives’ practices of client examination during postnatal care.

The findings revealed that less than three quarters of the midwives inspected perineal wounds (52.2%), checked vital signs of neonate (66.7%) and mother (62.2%), and inspected lochia drainage (30.4%) (Figure 2). Most midwives (91.3%) also reported that they never considered the emotional and psychological concerns of the mother when providing care (Figure 2). Conversely, most midwives reported that they did a head to toe examination of the neonate (82.2%) (Figure 2).

#### Health education and counselling

**FIGURE 3:**
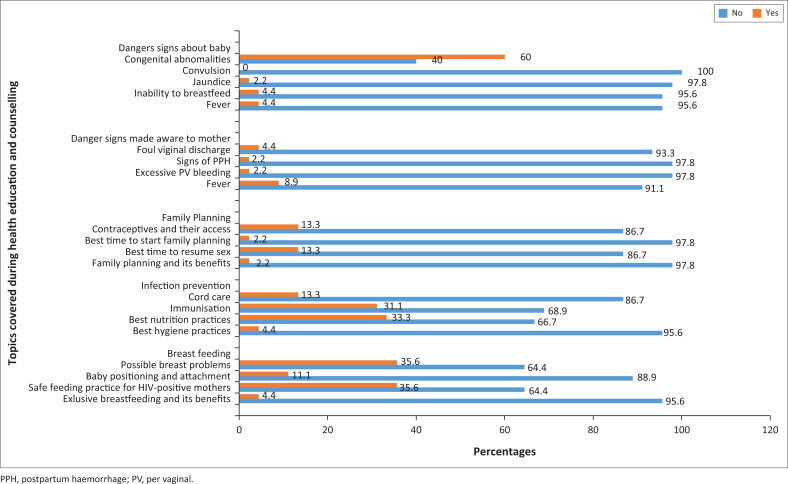
Health education and counselling topics covered by midwives during postnatal care.

The study found that all the midwives (100%) taught and counselled postnatal mothers during postnatal care and on discharge. Midwives covered a range of topics during health education and counselling (Figure 3). However, more than one third of midwives reported that they never taught the mother about congenital abnormalities being a danger sign to the baby (60%), immunisations (31.3%), best nutrition practices (33.3%), possible breast problems (35.6%) and safe feeding practices for HIV-positive mothers (35.6%) (Figure 3).

## Discussion

This study suggests that the quality of postnatal care received by mothers and their neonates was poor at all three health centres. The findings are corroborated by a previous study that reported the existence of substandard postnatal care in Malawi.^6^ In this study, the failure by some midwives to provide recommended interventions – including client monitoring, client examination, and education and counselling – negatively affected the quality of postnatal care. This may be attributed to a shortage of staff and the high midwife to client ratios (> 1:200) found in the three health facilities. There is evidence which showed that midwives offered substandard postnatal care at some health facilities in Malawi due to the shortage of staff.^6^ In addition, it has been documented that not all cadres of skilled birth attendants are able to offer the full range of EmOC to prevent maternal and newborn mortality and morbidity in Africa.^12^

It was clear from the findings of this study that some aspects of client examinations are not always accomplished by all midwives. For instance, in this study some of the midwives did not inspect mothers’ perineal wounds (47.8%), did not check vital signs of neonate (33.3%) and did not assess the emotional and psychological well-being of the mother (91.3%). This may increase the risk of poor quality postnatal care including midwifery misdiagnosis. However, the lack of resources such as a sphygmomanometer (21.7%), postnatal care guidelines (56.5%) and drugs (iron tablets = 60.9%, vitamin A = 52.2%) may have contributed to the poor quality of postnatal care rendered by the midwives in this study. This finding is in agreement with a study which found that many health facilities, especially in rural areas, experienced an intermittent supply of basic resources.^13^ The literature suggests that a lack of essential drugs and equipment has a negative impact on the quality of postnatal care^14^ in Malawi.^6,15^ This hampers efforts to reduce the maternal mortality rate^16^ in the country.

Despite all the midwives (100%) in this study reporting that they offered education and counselling to postnatal mothers, some midwives never covered certain aspects (Figure 3) including congenital abnormalities being a danger to the baby, immunisations and best nutrition practices. This may partly be attributed to the limited knowledge possessed by midwives. It has been documented that midwives lack knowledge on emerging issues in obstetrics in Malawi.^17^ Similarly, Dlamini and colleagues found that midwives in Botswana had serious gaps in knowledge and skills after undergoing in-service capacity-building training in EmONC and postnatal care.^18^ This supports calls for refresher training courses for midwives in maternal and neonatal health^6^ to equip them with current knowledge and skills, thus helping them to ably teach and counsel postnatal mothers. There should be adequate spacing of training in EmOC, retraining, and monitoring of training received to ensure compliance with standards for offering this care.

### Implications

Midwives are skilled attendants who provide postnatal care to the majority of mothers in Malawi. It is incumbent upon them to provide good quality postnatal care to mothers and newborns, as stipulated in prescribed guidelines. Midwives should effectively monitor and assess mothers and their babies. They should also provide relevant and adequate education and counselling to postnatal mothers.

### Limitations of this study

This study used a relatively small sample and results should be cautiously applied to other settings.

## Conclusion

This study has suggested that the postnatal care offered by midwives at three health facilities was generally substandard and midwives do not always monitor, assess and counsel postnatal clients. This negatively affects the quality of postnatal care received by mothers and their babies. The increased workload in these facilities highlights the fact that there should always be an adequate number of midwives to provide quality postnatal care in health facilities.
